# Hydrolyzed chicken extract (ProBeptigen®) on sleep quality in healthy individuals: a secondary analysis of PSQI global and component scores from a randomized double-blind trial

**DOI:** 10.3389/fnut.2026.1790576

**Published:** 2026-05-04

**Authors:** Lee Cheng Phua, Chaur-Jong Hu, Shan May Yong, Chin Chin Yau, Dean Wu

**Affiliations:** 1 Scientific Research, Suntory Beverage & Food Asia, Singapore, Singapore; 2 Department of Neurology, Shuang-Ho Hospital, Taipei Medical University, Taipei, Taiwan; 3 School of Medicine, College of Medicine, Taipei Medical University, Taipei, Taiwan; 4 PhD Program for Neural Regenerative Medicine, College of Medical Science and Technology, Taipei Medical University, Taipei, Taiwan

**Keywords:** clinical trial, hydrolyzed chicken extract, nutritional supplements, ProBeptigen®, sleep quality

## Abstract

**Background/objectives:**

Poor sleep is a prevalent health issue. This secondary analysis examined the effects of hydrolyzed chicken extract (ProBeptigen®) on sleep quality outcomes using data from a previously completed single-center, randomized, parallel, double-blind, placebo-controlled trial.

**Methods:**

Fifty-two healthy subjects with poor baseline sleep quality (Pittsburg Sleep Quality Index, PSQI global > 5) had been randomly assigned to receive either ProBeptigen® 670 mg (*n* = 24) or a placebo (*n* = 28) daily. Changes in PSQI total (global) score and individual component scores were analyzed.

**Results:**

Following 4 weeks of treatment, changes in PSQI global scores did not significantly differ between the two groups. However, the improvement in the subjective sleep quality component score was significantly greater in the ProBeptigen® group compared to the placebo group (*p* = 0.006). Additionally, subjects taking ProBeptigen® exhibited a numerically greater improvement in daytime function (*p* = 0.064).

**Conclusion:**

This proof-of-concept secondary analysis suggests that ProBeptigen® may improve perceived sleep quality and support further prospective evaluation.

## 1 Introduction

Poor sleep constitutes a major public health concern. According to population surveys, a high prevalence of sleep difficulties has been consistently reported across Asia. Specifically, 46.6% of the general population in Taiwan, 39.4% in Hong Kong, 26.4 to 31.1% in Japan, and 27.6% in Singapore reported poor sleep quality (1–4). The presence of sleep issues is strongly associated with a range of negative health outcomes, such as the development of diabetes mellitus, obesity, mood disorders and cardiovascular diseases, and reduced cognitive functions (5). Given the profound impact of this pervasive problem, effective interventions that improve sleep health are necessary.

Pharmacological treatments such as benzodiazepines, non-benzodiazepine sedative hypnotics and orexin receptor antagonists are available to manage sleep issues. However, the use of these treatments is associated with undesirable effects such as dependence, daytime sedation, and cognitive and psychomotor impairment (6). Nutritional supplements with more favorable side-effect profiles may represent a more suitable alternative and reflect a better benefit–risk trade-off for individuals with milder sleep problems. Yet, relatively few supplements with proven sleep benefits are available, thereby driving a need for further research.

In a recently published randomized controlled trial, hydrolyzed chicken extract (ProBeptigen®) was shown to improve short-term, long-term and working memory in middle-aged adults (7). Close to two-thirds of the subjects exhibited poor sleep status at baseline, characterized by a Pittsburg Sleep Quality Index (PSQI) global score of more than 5. In this study, we present a proof-of-concept secondary analysis to explore whether the sleep quality of these subjects (based on PSQI global and component scores) improved upon 4 weeks consumption of ProBeptigen®.

## 2 Methods

### 2.1 Study design and participants

We present a secondary analysis of data from a single-center, randomized, double-blind, placebo-controlled trial (7). Healthy adults aged between 35 and 65 years old with a body mass index (BMI) of 18.0 to 30.0 kg/m^2^ were enrolled in the trial. Participants were stratified by sex and randomly assigned to receive either 670 mg of ProBeptigen® every morning or a placebo, using a computer-generated randomization list provided by the sponsor, with sequential assignment at the study site. Participants, investigators, site staff, and outcome assessors remained blinded to group allocation throughout the trial. Data entry, management and verification were conducted by an independent data manager who was blinded to treatment assignment. The primary outcome was the change in cognitive function from baseline, with changes in sleep quality measured by PSQI among the secondary outcomes (7). In the current analysis, we focused on a subset of subjects with poor baseline sleep quality (PSQI global score > 5) and assessed changes in PSQI after 4 weeks of treatment, consistent with prior sleep studies (8, 9). The study was conducted according to the guidelines of the Declaration of Helsinki and approved by the Joint Institutional Review Board of Taipei Medical University (reference no. N201711060). All participants gave written informed consent prior to participating in the study.

### 2.2 Self-reported instruments

The 19-item Taiwanese PSQI assessed overall sleep quality and seven domains: subjective sleep quality, sleep latency, sleep duration, sleep efficiency, sleep disturbances, use of sleep medication and daytime dysfunction. Each PSQI component was scored from 0 to 3, with higher values reflecting greater impairment. The seven domain scores were summed to generate a PSQI total score ranging from 0 to 21, where higher scores denote poorer overall sleep quality. A total score above 5 is commonly used to indicate clinically relevant sleep disturbance (10). As each of the PSQI components assesses different sleep domains, examining both the global score and the individual components was useful in characterizing the intervention’s effects. The PSS, comprising 14 items, measured perceived stress, with higher scores indicating greater stress. The 21-item traditional Chinese Beck Depression Inventory-II (BDI-II) assessed depressive symptoms, with higher scores indicating more depressed mood.

### 2.3 Statistical methods

Changes in PSQI scores (global and component) from 0 to 4 weeks were compared between groups using a mixed model, with fixed effects for group, baseline score, age and sex. Analyses were conducted using complete-case data, including only participants with available outcome data. Model assumptions of residuals, including approximate normality, were assessed through visual inspection of Q–Q plots. The relationship between sleep quality and PSS or BDI was explored using Spearman’s rank correlation. For BDI, we used BDI-#16, which is BDI total score minus subitem 16 (sleep impairment related to depressive symptoms). A *p*-value <0.05 was considered significant.

## 3 Results

In the original randomized controlled trial, a total of 73 participants completed the trial with compatible drop-out rates of 16.3 and 21.3% in ProBeptigen® and placebo groups, respectively. No significant adverse events were reported and no drop-out was reported due to intolerance towards the treatment. Fifty-two participants who had a PSQI global score of more than 5 at baseline and repeated the test after 4 weeks treatment with either ProBeptigen® or placebo were included in the present analysis. All the patients performed PSQI, PSS, and BDI at baseline and after 4 weeks of therapy. Demographic and clinical data of the subjects were balanced between both groups at baseline (Supplementary Table S1).

Following 4 weeks of therapy, changes in PSQI global scores did not differ between the two groups (Table 1). However, the improvement in the subjective sleep quality component score (PSQI component 1) was significantly greater in individuals who consumed ProBeptigen® compared with those who took placebo (Table 1, *p* = 0.006). Additionally, subjects who took ProBeptigen® showed a numerically greater improvement in daytime function (PSQI component 7) compared to those who took placebo, although this difference did not reach statistical significance (Table 1, *p* = 0.064). No significant differences were observed in the remaining components.

**Table 1:** Changes in PSQI total and component scores from baseline to week 4 and between-group differences.

Variable	Changes from baseline to week 4		Between-group difference in change score (ProBeptigen® vs. placebo), 95% CI	*p*-value between groups
	ProBeptigen® (*n* = 24)	Placebo (*n* = 28)		
PSQI total score	−2.040 ± 0.584	−0.979 ± 0.553	−1.061 (−2.616 to 0.493)	0.176
PSQI Component 1: Subjective Sleep Quality	−0.500 ± 0.116	−0.062 ± 0.110	−0.438 (−0.748 to −0.129)	0.006*
PSQI Component 2: Sleep Latency	−0.306 ± 0.278	−0.235 ± 0.263	−0.071 (−0.810 to 0.667)	0.847
PSQI Component 3: Sleep Duration	−0.398 ± 0.173	−0.137 ± 0.165	−0.261 (−0.720 to 0.199)	0.259
PSQI Component 4: Sleep Efficiency	−0.320 ± 0.204	−0.087 ± 0.193	−0.233 (−0.776 to 0.310)	0.392
PSQI Component 5: Sleep Disturbances	−0.215 ± 0.102	−0.318 ± 0.096	0.103 (−0.171 to 0.377)	0.455
PSQI Component 6: Medicine usage	0.067 ± 0.075	−0.088 ± 0.071	0.155 (−0.044 to 0.354)	0.124
PSQI Component 7: Daytime dysfunction	−0.387 ± 0.135	−0.048 ± 0.128	−0.339 (−0.699 to 0.021)	0.064

Values are least square means (LSMs) ± standard error. Between-group differences represent LSM differences between groups (ProBeptigen® vs placebo) estimated from the linear mixed model, presented with 95% confidence intervals. *p < 0.05 (in bold).

Given the significant difference observed in the subjective sleep quality component score between the groups, a correlation analysis of the changes in this parameter was conducted with changes in PSS and BDI-#16. The change scores of BDI-#16 were significantly correlated with the change scores of subjective sleep quality (*R* = 0.435, *p* = 0.0013). There was also a small, non-significant positive correlation between the change scores of PSS and change scores of subjective sleep quality (*R* = 0.242, *p* = 0.0838).

## 4 Discussion

Sleep is a vital biological process essential for sustaining both physical and mental well-being. During sleep, the body repairs tissues, restores energy, consolidates memory, and removes neurotoxic waste (11, 12). Tools that support restorative sleep are hence crucial to enable better functioning during the day.

In this secondary subgroup analysis of a randomized, double-blind, parallel, placebo-controlled trial, hydrolyzed chicken extract (ProBeptigen®) significantly enhanced subjective sleep quality, as measured by PSQI component 1, in healthy adults with poor sleep at baseline. The mean improvement from baseline was greater with ProBeptigen® than with placebo (−0.500 versus −0.062), yielding a between-group difference of −0.438 points on the 0–3 scale. Although statistically significant, the absolute magnitude of improvement was modest and should be interpreted with caution. For context, a randomized placebo-controlled study of prolonged-release melatonin reported a similarly small absolute improvement in PSQI component 1 (approximately 0.2 points), suggesting that the magnitude observed here is consistent with other low-risk sleep aids (13). In contrast, established interventions such as cognitive behavioral therapy for insomnia generally produce substantially larger improvements in overall sleep outcomes, indicating that ProBeptigen® may be more appropriately considered a complementary rather than primary therapeutic approach. Beyond component 1, a trend towards better daytime functioning (PSQI component 7) was observed among those who took ProBeptigen®. However, other PSQI domains, including the PSQI global score, did not show statistically significant differences, potentially due to limited sample size. Given its good safety profile and the concurrent improvement of memory reported in the main analysis by Wu et al. (7), ProBeptigen® may represent a safe addition within a multifaceted sleep management strategy.

The improvement in the PSQI subjective sleep quality component score was significantly associated with changes in BDI scores and showed a trend toward association with PSS scores. While these exploratory correlations do not establish causal direction, they are consistent with broader literature describing close interrelationships between sleep and mood (14). As all measures were self-reported and collected at the same time points, shared method variance may partly account for these associations. Nevertheless, the concurrent shifts in sleep and mood indices highlight a potentially meaningful pattern that merits further investigation in studies specifically designed to examine these relationships.

Our findings align with previous data on protein hydrolysates. In a trial with Japanese participants suffering from insomnia, bovine alpha-S1 casein tryptic hydrolysate improved the PSQI global score and subjective sleep quality component score after 2 weeks, and sleep latency and daytime function after 4 weeks (8). Similarly, a 4-week study in Korean participants showed improvements in PSQI global score, and daytime functioning measured by the Epworth Sleepiness Scale (9). A 4-week fish hydrolysate supplementation also significantly enhanced the PSQI global score, and the component scores for subjective sleep quality, sleep efficacy, sleep disturbances, and daytime functioning (15). However, it is important to note that none of these studies found statistically significant differences between the supplement and placebo groups, indicating that the effects might be too marginal to surpass the placebo response. Indeed, across trials that reported PSQI Component 1 outcomes, between-group differences at approximately 4 weeks were negligible, ranging from −0.06 to 0 points. In contrast, the present study demonstrated a larger and statistically significant between-group difference (−0.438 points), suggesting that specific bioactive constituents in hydrolyzed chicken extract may contribute to a comparatively stronger improvement in perceived sleep quality.

The exact bioactives and mechanisms by which ProBeptigen® affects sleep quality remain unclear. ProBeptigen® contains various diketopiperazines produced through enzymatic and thermal treatments, and prior *in vivo* studies suggest that certain diketopiperazines may influence neurotransmitter systems such as GABAergic signaling or exhibit sedative effects (16, 17). It also contains tryptophan, a biochemical precursor of serotonin and melatonin, both of which are involved in well-characterized sleep regulatory pathways (18). However, these mechanistic pathways were not directly assessed in the present study. Further research is needed to clarify these hypotheses and identify the biological processes through which ProBeptigen® may influence sleep.

This study has several limitations. First, the small sample size may have reduced statistical power, limited generalizability, and increased susceptibility to random variation and false-positive findings. Consequently, the study may not have been adequately powered to thoroughly and reliably examine all components of the PSQI. Second, the analysis was *post hoc* and unadjusted for multiple comparisons, consistent with the exploratory nature of the analysis, and the findings should therefore be interpreted conservatively. Third, all sleep outcomes were based on self-reported PSQI measures. The lack of objective methods, such as actigraphy or polysomnography, prevented a comprehensive understanding of sleep profile changes. Nevertheless, the self-reported outcomes provided valuable insight into individuals’ overall sleep satisfaction, an important aspect of sleep management. Fourth, administering ProBeptigen® in the morning may not have been optimal for detecting sleep-related effects. If the supplement’s effects are time-dependent, evening administration may be more physiologically relevant and could enhance observable nighttime benefits. Future studies should therefore evaluate alternative dosing schedules, include larger sample sizes, and incorporate objective sleep measures. Finally, only the subjective sleep quality component showed improvement, and the magnitude of this effect was small. Thus, nutritional strategies such as ProBeptigen® should be considered as adjunctive interventions within a comprehensive strategy for supporting the overall sleep experience. Despite these limitations, the study’s strengths include its randomized, double-blind design and its role as the first proof-of-concept clinical investigation of hydrolyzed chicken extract’s effectiveness in improving sleep.

## 5 Conclusion

In conclusion, this study suggests that hydrolyzed chicken extract (ProBeptigen®) may improve perceived sleep quality, as reflected in PSQI component scores, among healthy adults with poor baseline sleep. Although no significant between-group differences emerged for PSQI global scores, improvements in specific components are encouraging and support further evaluation in larger, adequately powered trials. ProBeptigen® may serve as a supportive nutritional supplement within a broader, multi-pronged strategy for managing sleep health.

## Data Availability

The datasets presented in this article are not readily available due to ethical and privacy concerns. Requests to access the datasets should be directed to Dean Wu, tingyu02139@gmail.com.
