# Supplementary material for: Hydrolyzed chicken extract (ProBeptigen®) on sleep quality in healthy individuals: a secondary analysis of PSQI global and component scores from a randomized double-blind trial
Source: Front Nutr. 2026 May 4;13:1790576. doi: 10.3389/fnut.2026.1790576 (PMC13181736; doi:10.3389/fnut.2026.1790576)
Supplement: Supplementary file 1 [file Table_1.docx]

Table S1. Baseline demographic and clinical data of participants included in the analysis

|  | ProBeptigen (n=24) | Placebo (n=28) | *p*-value |
| --- | --- | --- | --- |
| Age, years | 41.79 ± 6.433 | 42.64 ± 7.093 | 0.655 |
| Sex, number (%) |  |  | 0.711 |
| Male | 8 (33.3%) | 8 (28.6%) |  |
| Female | 16 (66.7%) | 20 (71.4%) |  |
| PSQI total | 9.29 ± 2.758 | 9.57 ± 3.120 | 0.735 |
| PSQI component 1: Sleep quality | 1.79 ± 0.721 | 1.68 ± 0.670 | 0.560 |
| PSQI component 2: Sleep latency | 1.71 ± 1.042 | 1.64 ± 1.162 | 0.833 |
| PSQI component 3: Sleep duration | 1.58 ± 0.881 | 1.71 ± 1.084 | 0.638 |
| PSQI component 4: Sleep efficiency | 1.04 ± 1.122 | 1.21 ± 1.166 | 0.591 |
| PSQI component 5: Sleep Disturbance | 1.42 ± 0.504 | 1.64 ± 0.621 | 0.160 |
| PSQI component 6: Medicine usage | 0.29 ± 0.806 | 0.25 ± 0.701 | 0.843 |
| PSQI component 7: Daytime dysfunction | 1.46 ± 0.721 | 1.43 ± 0.790 | 0.888 |

Data were presented as mean ± standard deviation. *P*-values were obtained from the comparison between the ProBeptigen^®^ and placebo groups using the independent t-test for continuous variables and the chi-square (test for categorical variables)
